# The impact of stress on the behavior of C57BL/6 mice with liver injury: a comparative study

**DOI:** 10.3389/fnbeh.2024.1358964

**Published:** 2024-03-06

**Authors:** Mădălina Iuliana Mușat, Smaranda Ioana Mitran, Ion Udriștoiu, Carmen Valeria Albu, Bogdan Cătălin

**Affiliations:** ^1^U.M.F. Doctoral School Craiova, University of Medicine and Pharmacy of Craiova, Craiova, Romania; ^2^Experimental Research Centre for Normal and Pathological Aging, University of Medicine and Pharmacy of Craiova, Craiova, Romania; ^3^Department of Physiology, University of Medicine and Pharmacy of Craiova, Craiova, Romania; ^4^Department of Psychiatry, University of Medicine and Pharmacy, Craiova, Romania; ^5^Department of Neurology, University of Medicine and Pharmacy, Craiova, Romania

**Keywords:** anxiety, CUMS, depression, FSS, NAFLD

## Abstract

**Introduction:**

Depressive-like behavior has been shown to be associated with liver damage. This study aimed to evaluate the impact of three different models of depression on the behavior of mice with liver injury.

**Methods:**

During the 4 weeks of methionine/choline deficiency diet (MCD), adult C57BL/6 mice were randomly divided into four groups: MCD (no stress protocol, *n* = 6), chronic unpredictable mild stress (CUMS, *n* = 9), acute and repeated forced swim stress [aFSS (*n* = 9) and rFSS (*n* = 9)].

**Results:**

All depression protocols induced increased anhedonia and anxiety-like behavior compared to baseline and had no impact on the severity of liver damage, according to ultrasonography. However, different protocols evoked different overall behavior patterns. After the depressive-like behavior induction protocols, animals subjected to aFSS did not exhibit anxiety-like behavior differences compared to MCD animals, while mice subjected to CUMS showed additional weight loss compared to FSS animals. All tested protocols for inducing depressive-like behavior decreased the short-term memory of mice with liver damage, as assessed by the novel object recognition test (NORT).

**Discussion:**

Our results show that the use of all protocols seems to generate different levels of anxiety-like behavior, but only the depressive-like behavior induction procedures associate additional anhedonia and memory impairment in mice with liver injury.

## 1 Introduction

Not only does major depressive disorder remain an important global health problem (Friedrich, 2017), but it can also be associated with significant morbidity and mortality (Carney et al., 2002; Ramasubbu and Patten, 2003). Using basic experimental approaches, major breakthroughs have been made in the field. As such, the effectiveness of deep brain stimulation (Dournes et al., 2013), intranasal administration of transforming growth factor-β1 (Wei et al., 2021), or repetitive transcranial magnetic stimulation (Zuo et al., 2022) has been first shown to be effective in different animal models. This proved that the use of animal models in psychiatry can be used in other areas of research, in testing the efficacy of antidepressants, given their ability to mimic the symptomatology and etiology of depression (Zhang et al., 2015; Ayuob et al., 2016; Ali et al., 2017). Furthermore, animal experimentation has facilitated the identification of new molecular mechanisms (Munari et al., 2015; Verdonk et al., 2019), which may help in developing new targets for treatment-resistant depression.

However, as the field increased, inconsistencies became more obvious. For example, repeated forced swim stress (rFSS) is a well-known method for inducing depressive-like behavior in mice (Pesarico et al., 2020). Despite this, there are reports showing that swimming exercise may reverse depressive-like behavior induced by chronic unpredictable mild stress (CUMS) (Liu et al., 2018; Xie et al., 2022). While both FSS and CUMS are widely used protocols to study depressive-like behaviors in animal models, the forced swim stress paradigm focuses on acute stress and immobility as measures of depressive-like behavior (Au-Yankelevitch-Yahav et al., 2015), whereas the CUMS model involves chronic and unpredictable stressors (Burstein and Doron, 2018).

With the expansion of the field, more detailed and complex questions are put forward. As life expectancy increases, so does the likelihood that a patient will suffer from multiple diseases. From an animal model perspective, such overlapping pathologies generate an additional layer of complexity. For instance, in a large population-based study, it was observed that non-alcoholic fatty liver disease (NAFLD) was associated with the development of depression and anxiety disorders compared to matched controls without NAFLD (Labenz et al., 2020). However, most antidepressant treatments have variable cytotoxic effects (Elmorsy et al., 2017; Chen et al., 2020; Vijitkul et al., 2022). This means that testing the two pathologies in an animal model paradigm should use the most effective method to induce depressive-like behavior in animals, balancing the levels of anxiety that most of these models also imply (Anyan and Amir, 2018).

With NAFLD rapidly becoming a global epidemic (Setiawan et al., 2016; Murag et al., 2021), it is highly possible that researchers will use animal models of the two pathologies to better understand cell and system interactions. With several existing animal models of depression (Chourbaji et al., 2005; Der-Avakian et al., 2014) and NAFLD (Rinella et al., 2008; Jensen et al., 2018; Zhu et al., 2020), one will have to choose the best combination to best test their own hypothesis. Thus, it could be difficult to establish the optimal model required to examine the influence of depression and anxiety in animals that have a pre-existing hepatic injury. The present study aims to evaluate the impact of three distinct murine models of depression on the behavior of mice with liver damage and provide a clear description of the advantages and disadvantages associated with each model.

## 2 Materials and methods

### 2.1 Experimental animals

Experimental animals (16-18-weeks-old C57BL/6 male (*n* = 16) and female (*n* = 17), weighing 20–34 g) were housed in a room with a 12-h light/12-h dark cycle, controlled temperature (21–23°C), and humidity (60–70%), with food (provided in a food hopper) and water (standard autoclaved water bottle, 250 mL, 150 mm length, 55 mm diameter) freely available. The C57BL/6 mice were obtained from the Animal Facility of the University of Medicine and Pharmacy of Craiova. All experimental protocols and animal care were approved by the Committee for Experimental Animals Wellbeing of the University of Medicine and Pharmacy of Craiova (approvals no. 2.13 from 29.10.2020 and 2.1 from 10.11.2022).

### 2.2 Non-alcoholic fatty liver disease/non-alcoholic steatohepatitis induction

**Figure 1:**
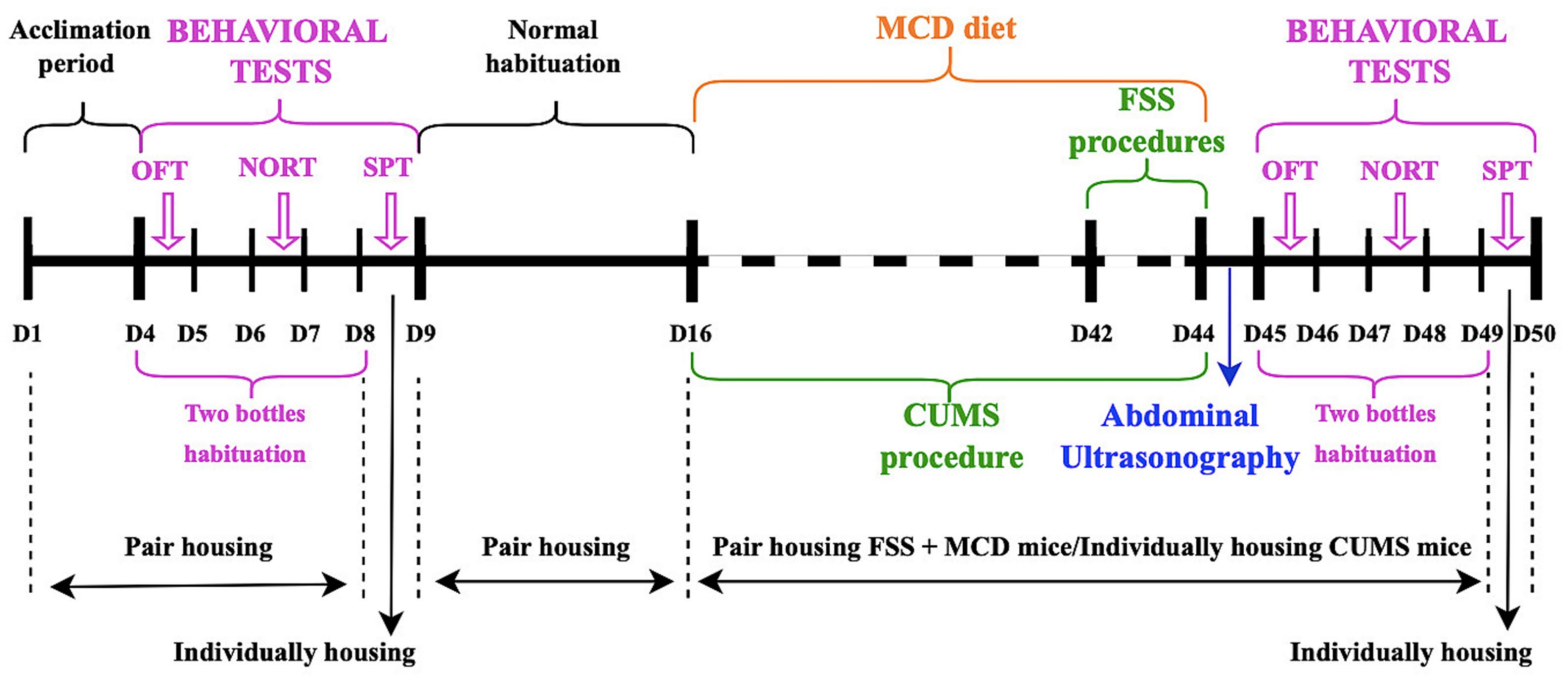
Time schedule for experimental design and mice housing.

All animals were allowed 3 days to acclimate to the new laboratory conditions before starting any experimental procedure. Non-alcoholic fatty liver disease/non-alcoholic steatohepatitis (Itagaki et al., 2013) was induced by replacing the normal food pellets with ones lacking methionine/choline food (MCD) (MP Biomedicals, Germany). All animals consumed the MCD food *ad libitum* for 4 weeks (Figure 1).

### 2.3 Depressive- and anxiety-like behavior induction

All animals were randomly divided into four groups: MCD (no stress protocol, *n* = 6; 3 males and 3 females), acute and repeated forced swim stress (aFSS (*n* = 9; 4 males and 5 females), rFSS (*n* = 9; 4 males and 5 females)) and CUMS (*n* = 9; 5 males and 4 females).

FSS procedures were performed using an open cylindrical container (30 cm height and 12 cm in diameter), with 20 cm of clear water (25 ± 1°C). Acute FSS animals were forced to swim for 10 min on two consecutive days (24 h apart). Following each exposure, mice were towel dried and placed into a new cage for 30 min to allow additional drying time before being returned to their home cages (Varlinskaya et al., 2020). Repeated FSS mice were forced to swim for a period of 15 min (day 1). After 24 h, the animals were again placed in water to swim through a sequence of four trials, each lasting 6 min. Between trials, mice were towel dried and returned to their home cage for 6 min (day 2) (Pesarico et al., 2020).

Mice assigned to the CUMS group were subjected to 28 days of repeated mild, unpredictable stressors (Wang et al., 2018; Herselman and Bobrovskaya, 2023; Li et al., 2023), one each day, with no repeated stressor within 3 days (Table 1).

**Table 1:** Schedule for CUMS procedure stressors by days.

Stressors	Duration	Days of CUMS procedure
Water deprivation	24 h	1, 14, 20, 27
Restraint stress in a tube	2 h	7, 13, 21
Continuous illumination	24 h	3, 9, 16, 22
Food deprivation	24 h	4, 10, 17, 23, 28
45° cage tilt	24 h	5, 11, 18, 25
Tail suspension	10 min	6, 12, 19, 26
Wet bedding	24 h	2, 8, 15, 24

During depressive- and anxiety-like behavior induction protocols, animals subjected only to the MCD diet and those from FSS groups were pair-housed (2–3 mice per cage) (Pesarico et al., 2020; Varlinskaya et al., 2020), while the animals undergoing the CUMS procedure resided in cages individually following the widely accepted scheme (Pałucha-Poniewiera et al., 2021). No disturbances, such as visitors or unrelated experimental procedures, were allowed in the animals’ room.

### 2.4 Clinical evaluation and behavior testing

Before starting any procedure, all mice were tested in order to establish a baseline. After the baseline tests, the animals were allowed 1 week of normal habituation before starting the experiments. The Sucrose Preference Test (SPT), Open Field Test (OFT) and Novel Object Recognition Test (NORT) were repeated at the end of all depressive-like behavior induction protocols. Behavioral tests were performed on different days, every day at the same hour (Figure 1). Before each behavioral test, the animals were allowed to acclimate to the testing room for 1 h. After each behavior trial, the testing surface was cleaned with 75% ethanol to remove odors. Body weight of the mice was measured weekly throughout the experiment.

SPT was used in order to assess anhedonia (Wu and Wang, 2010; Labenz et al., 2020). The SPT was performed based on a two-bottle choice paradigm, filled with 2% sucrose or tap water, with mice having free access to both for 24 h. Before testing, the mice were habituated to the presence of two drinking bottles for 4 days, and then the animals were deprived of food and water for 12 h. All animals were housed single per cage during the test. All the bottles have been checked, filled, weighed, and prepared before the test. The leaking ones have been replaced and carefully placed into the cages. Their positions were switched after 12 h to reduce any confounding produced by a side bias. After 24 h, we measured the volume of sucrose and water consumed and then calculated the animals’ affinity for sucrose. Sucrose preference is a percentage of the volume of sucrose consumption over the total fluid consumption during the test.

OFT was performed as previously described (Morega et al., 2021). Briefly, the test was carried out in an open arena [50 cm (length) × 33 cm (width) × 15 cm (height)]; the mice were placed individually in the center of the box, and the behavioral variables were recorded for 10 min and then analyzed (EthoVision XT 17, Noldus Technology). The amount of time the mouse spent in the center squares compared to the peripheral squares was used to measure anxiogenic behavior (Pitzer et al., 2019).

NORT was used to assess short-term memory (Toshkezi et al., 2018; Ahnstedt et al., 2020; Huo et al., 2021). One animal at a time was placed in an open field-like arena containing two identical objects and allowed to freely explore for 6 min. After that, the animal was returned, for 1 h, to its normal cage. The animal was then placed for another 6 min in the same arena. However, between the 2 sessions, one object was replaced by a new one. Using this second recording and an automatic system (EthoVision XT 17, Noldus Technology) the preference index was determined for each animal, as the percentage of time spent exploring the new object compared to the total time spent exploring both (Morega et al., 2022).

### 2.5 Abdominal ultrasonography

Hepatic ultrasound measurements were made using a S12-4 plane probe and a Philips CX50 Ultrasound Machine (Philips Healthcare, Netherlands). Ultrasound measurements were done after 4 weeks of MCD food. Parenchymal echotexture, nodules presence and the surface of the liver border were used for severity score (Table 2) (Morega et al., 2021). During the procedure, animals were anesthetized using a mixture of 1.5% Isoflurane and 49% O2 and 49% N2O via inhalation.

**Table 2:** Ultrasonography severity score.

Parameters	Criteria	Score
Parenchymal echotexture	Homogeneous, normal echotexture	0
	Heterogeneous echotexture	1
	Coarse echotexture	2
Nodule	None	0
	Micro nodule	1
	Macro nodule	2
	Micro and macro nodule	3
Surface or margin	Smooth	0
	Irregular	1

### 2.6 Statistical analysis

Statistical analysis was performed using GraphPad 9.4 and Microsoft Excel 2016. Differences in means among the groups were analyzed using one or two-way repetitive ANOVA (Tukey’s multiple comparisons test) with Geisser–Greenhouse correction, after the data set passed normality test (Shapiro–Wilk test and Kolmogorov–Smirnov test), and Kruskal-Wallis test (Dunn’s multiple comparations test) for non-parametric data. For the ANOVA test, Sessions (baseline and post-stress results) were used as a within-factor, and the four Protocols (MCD, aFSS, rFSS and CUMS) were considered as a between-factor. All figures show mean value and standard deviation (SD). Baseline and depression comparison use * *p* < 0.05, ** *p* < 0.01, *** *p* < 0.001 and **** *p* < 0.0001.

## 3 Results

### 3.1 Depressive and anxiety-like behaviors do not impact the severity of liver injury

**Figure 2:**
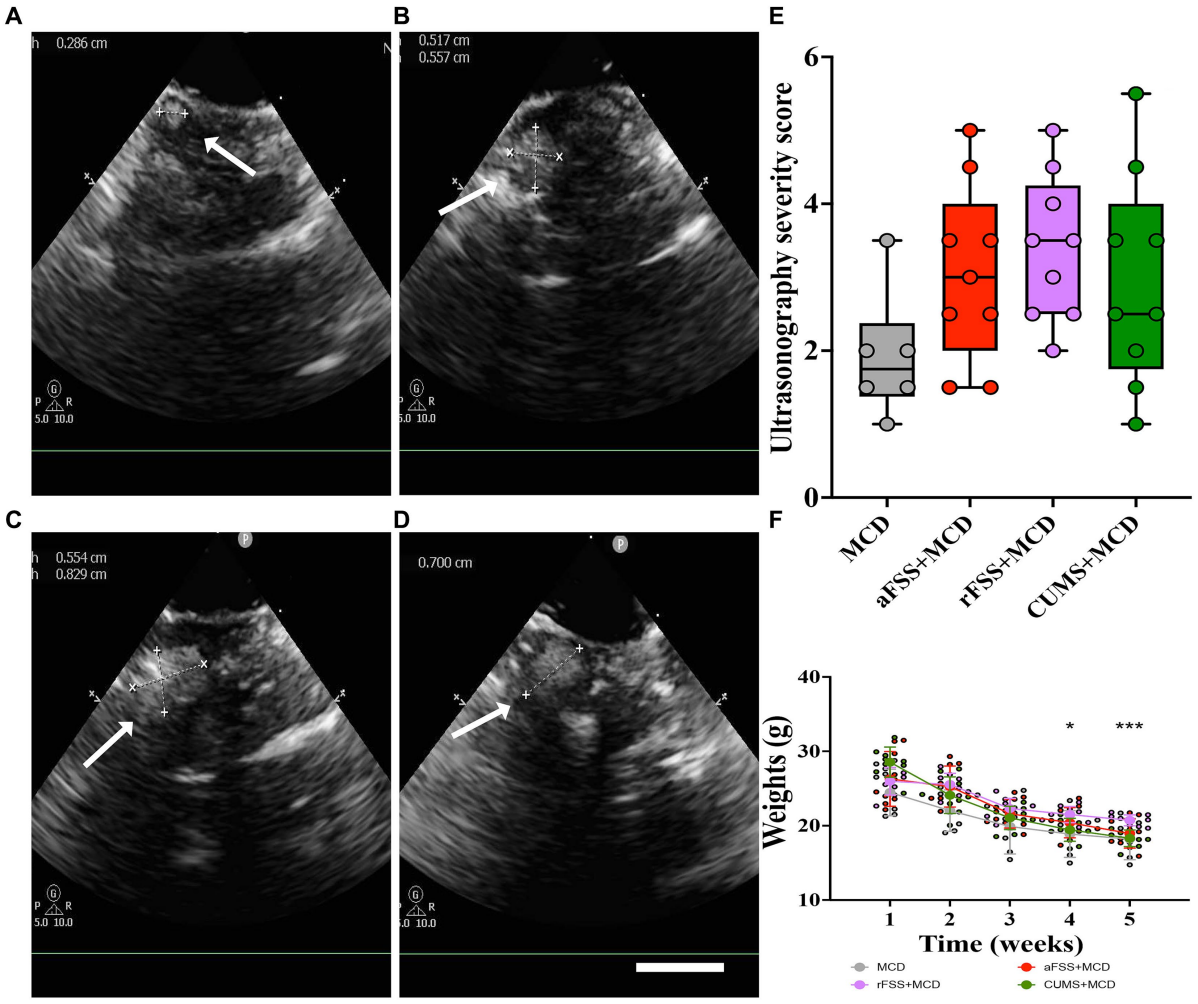
The assessment of animals with liver damage. Exemples of hepatic ultrasonography in **(A)** MCD group, **(B)** in aFSS, **(C)** rFSS and **(D)** in CUMS mice that showed the presence of various macronodules. **(E)** Ultrasonography severity score after 4 weeks of MCD diet showed no differences between the groups, although, the average severity score for mice subjected to aFSS and rFSS is higher compared to the MCD mice (3.05 ± 1.21, respectively, 3.38 ± 0. 99 compared to 1.91 ± 0.86). **(F)** Weekly measurements showed body mass loss for all investigated mice. Scale bar 2 mm. The graphs show mean values ± SD, * *p* < 0.05 and *** *p* = 0.0004.

The ultrasonography performed 4 weeks after the MCD diet was able to confirm the presence of liver damage in all animals fed the MCD diet (Figures 2A–D). Animals developed micro- and macro-nodules, with no differences in the severity scores between the groups (*p* > 0.05; Figure 2E).

When assessing the animals’ weight, the two-way ANOVA revealed variances between Sessions (F_1.744,50.58_ = 433.1, *p* < 0.0001). All animals lost body mass during the experiment, regardless of Protocols. Although no differences were observed in the utilized Protocols (F_3,29_ = 1.780, *p* = 0.1730), significant Interaction was noticed between Sessions and Protocols (F_12,116_ = 11.05, *p* < 0.0001). Post-hoc test revealed no differences between Protocols in baseline. From baseline to post-stress session, all animals from MCD (*p* = 0.0002), aFSS (*p* < 0.0001), rFSS (*p* < 0.0001) and CUMS (*p* < 0.0001) groups displayed a reduced weight. After MCD diet and post-stress, CUMS mice exhibited a lower weight compared to rFSS group, starting with the 4^th^ week of the experiment (*p* = 0.0243) and in week 5 (*p* = 0.0004) (Figure 2F).

### 3.2 Repeated FSS and CUMS induce increased anhedonia in mice with liver damage

**Figure 3:**
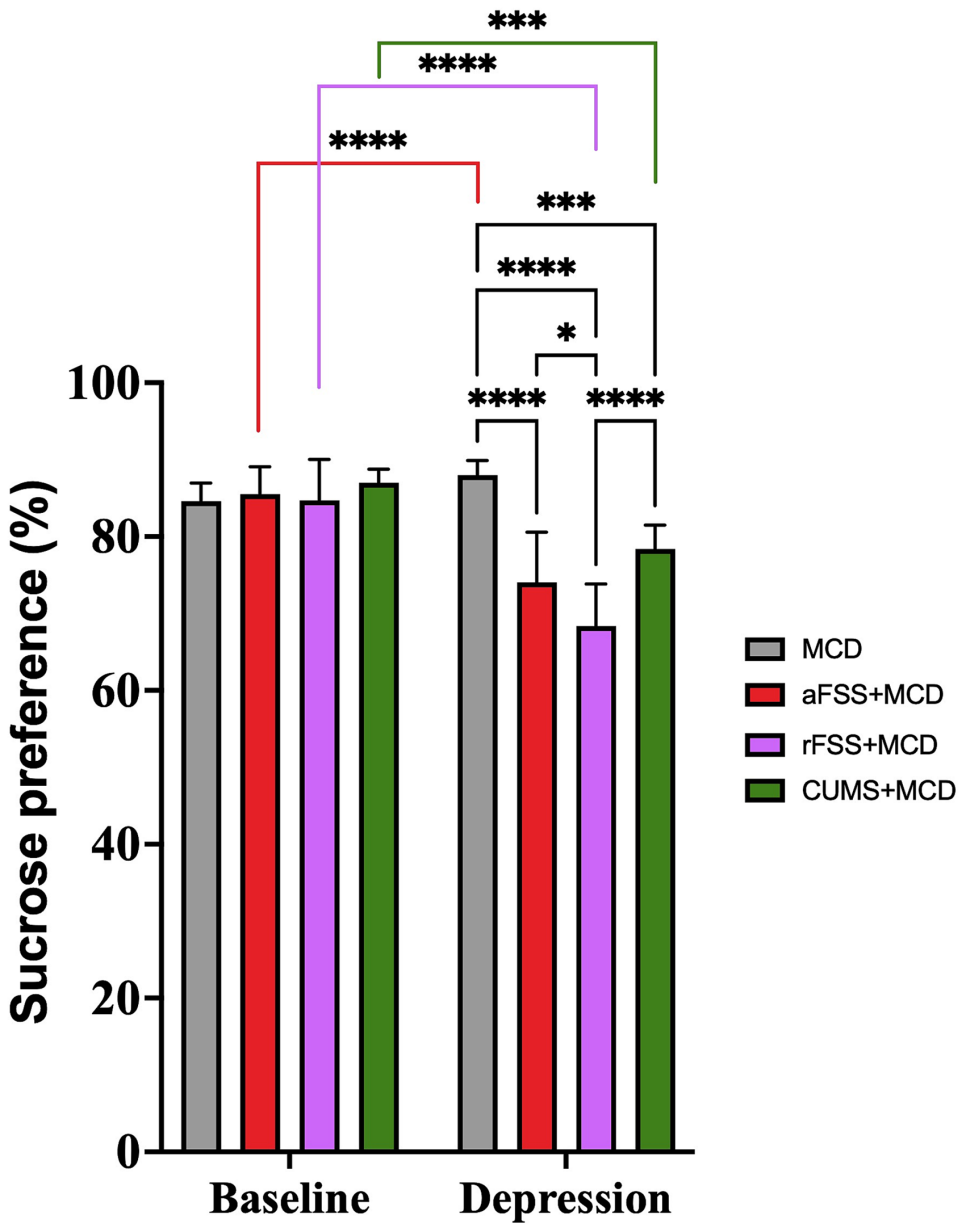
Anhedonia-like behavior of mice with liver damage. After 4 weeks of the MCD food, there was no difference in the level of anhedonia in mice that were not subjected to any depressive-like behavior induced procedures (*p* = 0.445). All other investigated groups developed anhedonia-like behavior compared to baseline. Mice subjected to rFSS displayed the lowest sucrose preference, and significantly lower (68.36 ± 5.47%) compared to the aFSS (74.05 ± 6.51%, *p* = 0.037) and CUMS group (78.39 ± 3.08%, *p* < 0.0001). The graph shows mean values ± SD, * *p* < 0.05, **** *p* < 0.0001.

The use of ANOVA in order to evaluate the anhedonia revealed differences between Sessions (F_1,29_ = 73,72, *p* < 0.0001). Anhedonia increased in all animals subjected to stress, regardless of the Protocol used. ANOVA also revealed significant differences between Protocols (F_3,29_ = 11.90, *p* < 0.0001). Post-hoc assessment of sucrose preference revealed that animals subjected to rFSS displayed the lowest sucrose preference compared to CUMS (*p* < 0.00001) and aFSS group (*p* = 0.037) (Figure 3).

The Interaction was also significant (F_3,29_ = 16.46, *p* < 0.0001). Post-hoc test revealed that there were no differences between Protocols in baseline. All groups, except MCD (*p* = 0.445), decreased the sucrose preference from baseline to post-stress session. Animals subjected to aFSS procedure displayed a 74.05 ± 6.51% preference compared to their baseline value of 85.51 ± 3.56% (*p* < 0.00001). The number dropped from 84.71 ± 5.30% at baseline to 68.36 ± 5.47% (*p* < 0.0001) for animals subjected to rFSS procedure. Mice subjected to CUMS protocol also showed a reduced preference for sucrose (78.39 ± 3.08%), compared to baseline (87.07 ± 1.77%) (*p* = 0.0002) (Figure 3). In post-stress session, mice from aFSS (*p* < 0.0001), rFSS (*p* < 0.0001) and CUMS (*p* = 0.0005) groups displayed increased anhedonia compared to MCD animals.

### 3.3 All depressive models exacerbate anxiety-like behavior in MCD Fed mice

**Figure 4:**
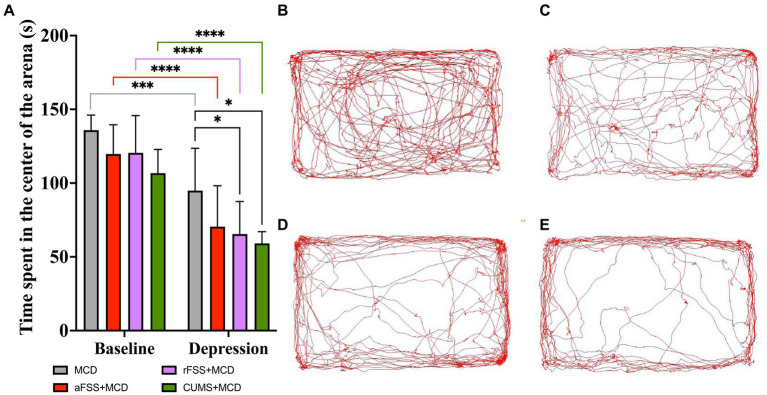
Animal anxiety-like behavior assessed by OFT. **(A)** While MCD fed animals display change in anxiety-like behavior (*p =* 0.0001), rFSS and CUMS exacerbate the MCD anxiety-like behavior. Analyzing the recorded arenas and tracking the mice’s path revealed that animals subjected to **(B)** MCD, **(C)** aFSS, **(D)** rFSS and **(E)** CUMS, after 4 weeks of MCD diet, displayed different anxiety-like behavior levels. **(B–E)** shows examples of the path and tracking of individual mice recorded inside the arena, as representative of their respective group. The graph shows mean values ± SD, * *p* < 0.05, *** *p* < 0.001, **** *p* < 0.0001.

Two-way ANOVA performed in order to assess the anxiety-like behavior revealed differences between Sessions (F_1,29_ = 176.0 *p* < 0.0001). Regardless of the Protocols, all animals displayed a decrease in the time spent in the center of the arena. Regarding Protocols significant differences were observed (F_3,29_ = 3.847, *p* = 0.0197), but no effect in Interaction (F_3,29_ = 0.5715, *p* = 0.6383). Testing at baseline revealed no differences between groups. From baseline to post-stress session, all animals exhibited a decrease in time spent in the center of the arena. Acute FSS induced a decrease to 70.49 ± 27.66 s compared to 119.72 ± 19.86 s at baseline (*p* < 0.0001). Repeated FSS decreased this interval to 65.45 ± 22.08 s compared to 120.47 ± 25.28 s (*p* < 0.0001) (Figure 4A). Animals subjected to CUMS procedure manifested increased anxiety-like behavior, spending the least amount of time in the center of the arena (59.01 ± 8.03 s), compared to baseline (106.69 ± 16.06 s) (*p* < 0.0001), and MCD animals explored only for 94.88 ± 28.73 s compared to baseline, where they explored for 135.89 ± 10.23 s (*p* = 0.0001) (Figure 4A). In post-stress session, animals subjected to rFSS and CUMS procedures manifested additional increases in anxiety-like behavior, spending less time in the center of the arena (65.45 ± 27.08 s, respectively 59.01 ± 8.03 s), compared to mice from MCD group (94.88 ± 28.73 s) (*p* = 0.046 and *p* = 0.009). No differences were observed in the levels of anxiety-like behavior in animals subjected to aFSS protocol, compared to mice from MCD group (*p* = 0.133) (Figure 4A).

The tracking paths of the mice during the test were illustrated by one representative animal from each group: MCD (Figure 4B), aFSS (Figure 4C), rFSS (Figure 4D), and CUMS (Figure 4E).

### 3.4 All depressive models decrease short-term memory in mice Fed the MCD diet

**Figure 5:**
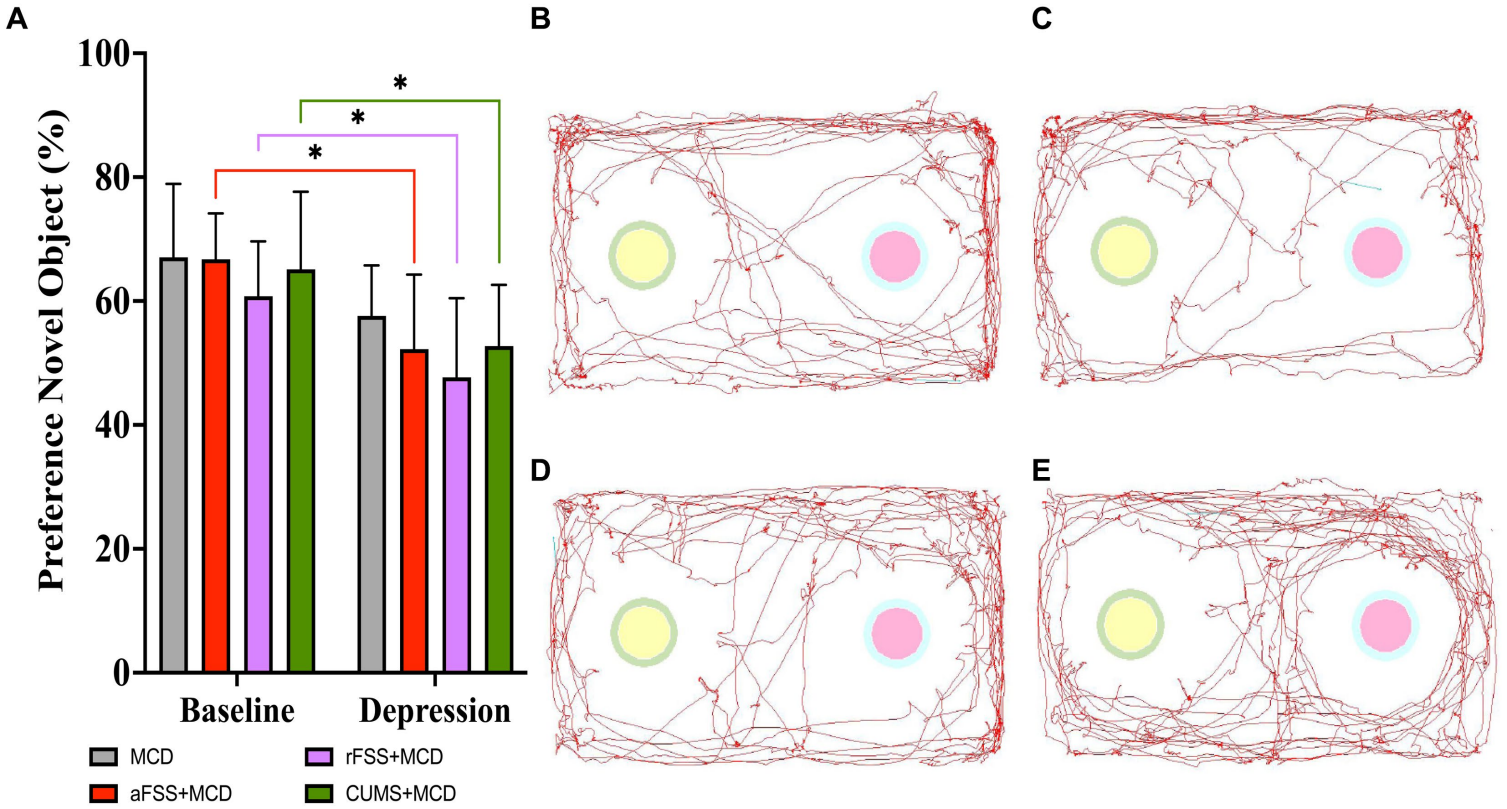
Short-term memory assessment using NORT. **(A)** All animals subjected to depressive-like behavior induced protocols showed a heightened discrimination index of 52.22 ± 12.05% (*p* = 0.010) for aFSS, 47.68 ± 12.77% (*p* = 0.022) for rFSS and of 52.73 ± 9.89% (*p* = 0.033) for CUMS animals and displayed a worse average discrimination index after stress protocols, but no difference in short-term memory was observed between baseline and post-stress in MCD group. The animal’s preference for the novel object was illustrated by the track path of one mouse from each group: **(B)** MCD, **(C)** aFSS, **(D)** rFSS, and **(E)** CUMS. The graph shows mean values ± SD, * *p* < 0.05.

The two-way ANOVA showed differences between Sessions (F_1,29_ = 28,18, *p* < 0.0001). Regardless of Protocols, the preference index for novel object decreased in all stressed animals. No differences were observed in Protocols (F_3,29_ = 1.240, *p* = 0.3131) and Interactions (F_3,29_ = 0.1806, *p* = 0.9087). Testing at baseline revealed no differences between groups. From baseline to post-stress session, all groups, except MCD (*p* = 0.310), displayed a decreased in short-term memory. Acute FSS animals dropped their index to 52.22 ± 12.05% (*p* = 0.010), repeated FSS to 47.68 ± 12.77% (*p* = 0.022) and CUMS group to 52.73 ± 9.89% (*p* = 0.033) (Figure 5A). No difference between groups in post-stress session were observed.

The animals’ preference for the novel object recorded inside the arena was displayed by one representative mouse from each group: MCD (Figure 5B), aFSS (Figure 5C), rFSS (Figure 5D), and CUMS (Figure 5E).

## 4 Discussion

The correlation between depression and NAFLD has been established through clinical (Labenz et al., 2020; Cho et al., 2021) and experimental evidence (Zhu et al., 2020). Here, we tested three depressive-like behavior induction paradigms on mice with NAFLD and evaluated mice levels of depressive and anxiety-like behavior. NAFLD was quantified using ultrasound evaluation, a safe, non-invasive, and very well tolerated method (Wong et al., 2018). Widely recognized as the most commonly used method of inducing NAFLD in rodents (Jorgačević et al., 2014; Kim et al., 2017), the MCD diet has been reported to lead to notable weight loss (Ning et al., 2020), development of liver nodules, increased echogenicity and inflammation assessed by ultrasonography in mice (Brzezinski et al., 2020), hepatic changes that have also been associated with NAFLD in humans (Khov et al., 2014; Yang et al., 2019). Although the average severity scores for our mice subjected to aFSS, rFSS and CUMS are higher compared to the MCD mice, no significant difference was observed. While depressive-like behavior was reported in models of chronic liver injury caused by intraperitoneal injection of CCl_4_ and D-galactosamine (Zhu et al., 2020), in the present study we were not able to show a similar trend in MCD-fed animals without any depressive-like behavior inducing protocols. This could mean that alterations in behavior resulting from liver damage may be contingent upon the degree of liver damage, as the use of intraperitoneal injection of CCl_4_ induces necrosis, swelling, loose cytoplasm and inflammatory infiltrate, D-galactosamine was reported to induce scattered inflammatory infiltration and hepatic sinusoid hyperemia (Zhu et al., 2020), and MCD induces steatosis with intra-lobar diffuse inflammation (Morega et al., 2021). Except for some minor behavioral differences, most of the studies do not report the sex of C57BL/6 mice as a determining factor for varied outcomes following liver injury (Zhu et al., 2020). This is interesting as traditionally, due to hormone cycles differences (Romeo et al., 2003; Maguire et al., 2005), there has been a long-lasting concern in animal research regarding the use of both females and males, and as such, in basic research, the sole use of male animals was recommended (Shansky, 2019). However, the inclusion of gender as a biological variable has encouraged researchers to use both male and female animals in fundamental research (Clayton and Collins, 2014). Surprisingly, in neuroscience, the inclusion of females did not increase variability in rodent research (Beery, 2018), but it introduced new experiments that were needed in order to justify broader conclusions (Frick et al., 2000). As we become aware that frequently used tests can generate behavioral differences between genders (Meziane et al., 2007; Nisbett et al., 2023), depending on the question asked, that particular test can be avoided. As such, in the present study, we were careful to avoid using tail suspension test to investigate depressive-like behavior in mice, as it is known to have gender differences (Tsao et al., 2023). Furthermore, traditional concerns regarding behavioral differences in female animals due to estrous cycle were shown to have minimal impact (Zeng et al., 2023). In order to mitigate differences in gender and estrous cycle, we opted to use C57BL/6 mice and we used OFT and the more laborious SPT (Meziane et al., 2007; Tsao et al., 2023; Zeng et al., 2023).

With all used procedures already applied to healthy animals, in various approaches and different species of rodents, proving their validity and showing that both FSS (McLaughlin et al., 2003; Pesarico et al., 2020) and CUMS (Herselman and Bobrovskaya, 2023; Li et al., 2023) are inducers of heightened anxiety-like behavior in healthy animals (Mineur et al., 2006; Sequeira-Cordero et al., 2019), here we were able to show that these procedures are also effective on the MCD fed mice. However, some behavioral differences were observed between animals with liver damage subjected to these procedures. While liver damage in humans can be associated with depression and anxiety disorders compared to matched controls without NAFLD (Labenz et al., 2020), mice fed MCD food did not display increased anhedonia compared to baseline, but they did have an increased anxiety-like behavior with no additional inducing procedure. This confirmed prior research showing that as little as 3 weeks of the MCD food can trigger alterations in anxiety-like behavior levels in younger mice, individually housed, as observed by OFT (Morega et al., 2022). Despite the heighten anxiety-like behavior of the MCD animals, both rFSS and CUMS protocols significantly increased animal anxiety-like behavior. The use of aFSS did not generate any additional anxiety-like behavior, as aFSS animals did not display increased anxiety-like behavior compared to MCD animals. The observed phenomenon may be attributed to the use of a different rodent species or of mature animals, thereby corroborating previous findings that anxiety is induced only in juvenile subjects rather than in adults under aFSS protocol (Varlinskaya et al., 2020).

Subjecting MCD mice to procedures that induce depressive-like behavior determined an increased in anhedonia, with rFSS having the worst anhedonic outcome. The sucrose preference in our CUMS mice was significantly lower compared to baseline, but higher than reported in other studies. This difference may be due to the higher number of stressors used (Li et al., 2023) or the total duration of the CUMS procedure (Alqurashi et al., 2022). As such, we speculate that the anhedonia outcome, as recorded by the SPT, might be contingent on both the duration of the stressful period and the number of stressors involved. Furthermore, when performing the SPT, one should always take into consideration that the preference for sucrose is dependent on the animal’s capability to respond to natural reward (Sequeira-Cordero et al., 2019).

We were able to partially confirm prior reports that have indicated a loss in short-term memory after CUMS (Leo et al., 2021), by observing a decrease in the discrimination index compared to baseline. However, this decrease is not greater than MCD control animals, showing that, in this case, there might be an additional factor to consider, since all depressive-like behavior inducing protocols generated similar results. It remains unclear whether the observed memory impairments are generated by the liver damage, or by the experimental method used to induce it, as neuronal loss has been reported in animals fed MCD diet (Morega et al., 2022).

When conducting similar studies, ethical problems should be considered, as both CUMS protocol (Jiao et al., 2021) and MCD diet (Morega et al., 2021) were reported to frequently reduce body mass in mice. In the present study, animals subjected to the CUMS procedure displayed the lowest weight and had an overall worse general state, especially compared to those subjected to FSS protocols. This may suggest that chronic stressors have a more profound impact on animal health than acute ones, as auto-grooming behavior is highly sensitive to stress (Kalueff and Tuohimaa, 2004) and may be indicative of a disturbance in self-directed behavior (Nollet, 2021). Future investigations employing this procedure should prioritize the animal’s condition, particularly when considering aged subjects, as the impact could be heightened. Aged subjects may be more susceptible to the negative consequences of chronic stress due to age-related changes in physiological resilience and adaptive capacity (Kirkland et al., 2016). Therefore, if other studies would be interested in aged groups, the experimental designs should include careful monitoring of the health status of aged animals during chronic stress procedures. Although aggression in group-housed mice is common (Weber et al., 2017), mice are social animals (Van Loo et al., 2004) and individual housing causes additional stress to mice (Kalliokoski et al., 2014). Future studies can explore and evaluate alternative housing strategies for mice to reduce aggression while still allowing social interactions. This could involve designing more enriched and spacious group housing environments that provide opportunities for mice to establish territories and engage in natural social behaviors. However, one should take into account that these could lead to a lower effectiveness of the CUMS protocol. The isolation represents a housing approach frequently encountered in CUMS procedure (Pałucha-Poniewiera et al., 2021), with no mention of it in the FSS protocol (McLaughlin et al., 2003; Pesarico et al., 2020). In order to ensure that our findings remained unaffected by pair or individually housing, we avoided using forced swim and tail suspension as behavioral tests, taking into account that group housing of mice increases immobility in these tests (Karolewicz and Paul, 2001). Moreover, different stressors can be applied as part of the CUMS paradigm (Nollet, 2021; Pałucha-Poniewiera et al., 2021). However, in our opinion, when choosing sever stressors (painful stress, water and/or food deprivation) more behavior tests should be considered to correctly interpret the results.

Several studies have demonstrated that acute (Yamanishi et al., 2022) and chronic (Du Preez et al., 2021) stressors, as well as a MCD diet (Toshkezi et al., 2018; Varlinskaya et al., 2020), can significantly affect the cell populations of the central nervous system. Therefore, experimental research involving both stressors and an MCD diet should exercise caution in selecting appropriate controls, as multiple factors, including neuroinflammatory responses, are involved in the pathogenesis of depression. For instance, microglia-mediated neuroinflammation is essential for the development of depression (Yirmiya et al., 2015), with microglial activation observed in depressed patients who committed suicide (Steiner et al., 2008). One of the most important limitations of the present study is the absence of cellular evaluations, while the relatively low number of animals investigated could change borderline behavior results. As such, although important to our understanding of pathology, animal models pose a translational question that needs to be carefully answered.

## 5 Conclusion

The rising prevalence of psychiatric disorders has led to a growing demand for reliable animal models that can aid further scientific investigation. The co-occurrence of depression with multiple comorbidities is now a common phenomenon. According to our findings, the sole use of MCD diet can lead to anxiety-like behavior in young C57BL/6 mice. However, the use of depressive models additionally decreased short-term memory and increased anhedonia in mice fed the MCD diet, with repeated FSS showing the lowest sucrose preference in the tested animals. The severity of liver injury in our mice remained unaffected by the depressive and anxiety-like behavior induction protocols used.

## Data availability statement

The raw data supporting the conclusions of this article will be made available by the authors, without undue reservation.

## Ethics statement

The animal study was approved by the Committee for Experimental Animals Wellbeing of the University of Medicine and Pharmacy of Craiova (approvals no 2.13 from 29.10.2020 and 2.1 from 10.11.2022). The study was conducted in accordance with the local legislation and institutional requirements.

## Author contributions

MM: Data curation, Formal analysis, Investigation, Methodology, Writing – original draft. SM: Investigation, Methodology, Writing – original draft. IU: Supervision, Writing – review & editing. CA: Supervision, Writing – review & editing. BC: Formal analysis, Methodology, Resources, Supervision, Validation, Writing – review & editing.
